# Carbon Dot-Mediated Photodynamic Treatment Improves the Quality Attributes of Post-Harvest Goji Berries (*Lycium barbarum* L.) via Regulating the Antioxidant System

**DOI:** 10.3390/foods13060955

**Published:** 2024-03-21

**Authors:** Juan Du, Zhi-Jing Ni, Wei Wang, Kiran Thakur, Run-Hui Ma, Wen-Ping Ma, Zhao-Jun Wei

**Affiliations:** 1 School of Biological Science and Engineering, North Minzu University, Yinchuan 750021, China; 20217528@stu.nun.edu.cn (J.D.); lovebear@vip.163.com (Z.-J.N.); wwang@nun.edu.cn (W.W.); kumarikiran@hfut.edu.cn (K.T.); zhantingbaiyang@163.com (R.-H.M.); 2 School of Food and Biological Engineering, Hefei University of Technology, Hefei 230009, China

**Keywords:** goji berry, carbon dots-mediated photodynamic treatment, antioxidant enzymes, post-harvest senescence

## Abstract

Carbon dots (CDs) have been proposed as photosensitizers in photodynamic treatment (PDT), owing to their excellent biological attributes and budding fruit preservation applications. In the present study, CDs (4.66 nm) were synthesized for photodynamic treatment to improve the quality attributes in post-harvest goji berries. The prepared CDs extended the storage time of the post-harvest goji berries by 9 d. The CD-mediated PDT postponed the hardness and decay index loss, reduced the formation of malondialdehyde (MDA), hydrogen peroxide (H_2_O_2_), and superoxide anion (O_2_^•−^) significantly, and delayed the loss of vital nutrients like the total protein, phenols, and flavonoids. The CD-mediated PDT improved the catalase (CAT), ascorbate peroxidase (APX), peroxidase (POD), phenylalanine ammonia-lyase (PAL), glutathione reductase (GR), and superoxide dismutase (SOD) activities, but did not improve polyphenol oxidase (PPO) activity. In addition, The CD-mediated PDT induced the accumulation of ascorbic acid (ASA) and glutathione (GSH). Overall, a CD-mediated PDT could extend the storage time and augment the quality attributes in post-harvest fresh goji berries by regulating the antioxidant system.

## 1. Introduction

Goji (*Lycium barbarum* L.), a kind of oval orange-red berry that originates from Asia, mainly grows in arid and semi-arid areas. Its rich functional composition (polysaccharide, carotenoid, and betaine) makes it an ideal food and drug resource that can increase the human metabolism and regulate human immune functions [1]. At present, research reports mainly focus on the roles of chemical preservatives and diverse packaging strategies to improve the life and quality of stored fruit, including hydrogen sulfide fumigation [2], salicylic acid treatment [3], nitric oxide soaking treatment [4], heat treatment combined with chitosan coating [5], and lotus leaf extract composite coating [6]. The present application preservation technologies of fresh goji berries pose environmental challenges and lead to an accumulation of chemical residues. Therefore, more novel preservation technologies are needed for the further development of the goji berry industry and its economic value.

In recent years, photodynamic treatment (PDT) based on a photosensitizer (PS) has emerged as an important food preservation method due to its innocuity, low cost, and low energy consumption, and its use does not change the substrate, nutritional value, and sensory quality of the food or produce harmful products [7].The basic principle of PDT is that ROS are generated by a photochemical reaction that is initiated by the combination of visible light, photosensitizer compounds, and molecular oxygen. This reaction causes severe oxidative damage to microbial cells [8]. ROS are formed through type I reactions, which produce hydroxyl radicals (•OH), superoxides (O_2_^•−^), or hydrogen peroxide (H_2_O_2_), or through type II reactions, which produce singlet oxygen (^1^O_2_) [9]. It is important to note that photosensitizers (PSs) play a crucial role in improving the efficacy and safety of a PDT [10]. The current research on PSs mainly focuses on natural PSs, such as riboflavin [11], curcumin [12], and hypericin [13]. A natural PS is characterized by the low in vivo toxicity and high removal efficiency but exhibits a poor water solubility and unstable chemical properties [14]. To solve this problem, carbon dots (CDs), with their simple synthesis method, low cost, and environmental protection, have attracted more and more attention as a photosensitizer for PDT [15]. Nied et al. conducted a comprehensive study on the use of carbon dots as a photosensitizer for antibacterial photodynamic therapy in vitro. The experiment involved synthesizing carbon dots using citric acid and ethanol as the environmental protection solvents. The carbon dots were then irradiated with a blue light LED, which resulted in the generation of singlet oxygen that effectively inactivated Escherichia coli and Staphylococcus aureus. The study found that CDs act through a type II mechanism and are an effective photosensitizer for PDT [16]. Therefore, CDs have great potential as a photosensitizer for PDT in the future of food preservation.

Compared with traditional semiconductor quantum dots, CDs exhibit good water solubility, biocompatibility, antibacterial properties, and unique physical and chemical properties, supporting their high application value mainly in biological imaging and biosensors [17]. With the burgeoning research interest in CDs, researchers have developed various synthesis methods, broadly categorized into “top–down” [18] and “bottom–up” approaches [19]. Top–down methods, such as laser ablation, acid-assisted chemical oxidation, or electro-oxidation, require intricate synthesis conditions. In contrast, bottom–up methods offer simplicity and ease of operation. The hydrothermal and microwave methods are notable for their simple equipment requirements, controllable synthesis parameters, and high efficiency. The CDs synthesized using these methods have a uniform particle size distribution, making them suitable for large-scale production [20,21]. As a result, they have received significant attention from researchers.

The choice of carbon precursor has a significant impact on the performance of CDs. Considerable research has been conducted on using affordable chemicals or natural materials as the precursors for CD synthesis [22]. The CDs extracted from kelp by Kai Fan et al. [23] have been applied in combination with chitosan (CH) to preserve fresh-cut cucumbers. The results showed that the CD/CH coating could inhibit microbial reproduction, thus ensuring the quality of the cucumbers and extending their shelf life. Riahi et al. [24] synthesized chitosan-based CDs with multiple functions by using the hydrothermal method, and they displayed a strong antioxidant activity and strong antibacterial activity against *Aspergillus niger* and *Aspergillus flavus*. However, natural precursors are not suitable for large-scale production due to their high synthesis cost, uneven heating, time consumption, and low quantum yield [22]. Furthermore, the antioxidant ability of the CDs may be regulated by the antioxidant ability of their precursors or related to specific functional groups, such as carbon–carbon double bonds (C=C), special elements, and other factors [25]. In contrast, the CDs synthesized using the hydrothermal method with citric acid and ethylenediamine as the precursors have several advantages, including a mature synthesis technology, simple reaction conditions, short reaction time, and the use of cheap and readily available raw materials. The high quantum yield and stable fluorescence performance of the CDs synthesized using these two raw materials have been demonstrated in Zhu et al.’s research [26]. Based on these studies, citric acid is identified as a food additive with antioxidant properties, and ethylenediamine is noted for its functional group (NH_2_). Thus, the CDs synthesized through the hydrothermal method, using citric acid and ethylenediamine as the precursors, are anticipated to have wide-ranging application prospects.

In this study, citric acid and ethylenediamine were used to synthesize CDs, which were then used as the PS of PDT in the preservation of post-harvest fresh goji berries. Then, the effects of light irradiation, illumination time, and PS concentration on the preservation of post-harvest fresh goji berries were discussed, and the influences of various indexes on the storage quality of post-harvest fresh goji berries were revealed. This study provided new technical methods and ideas for the application of CD-mediated photodynamic treatment in the preservation of fresh goji berries.

## 2. Materials and Methods

### 2.1. Preparation and Characterization of Carbon Dots (CDs)

**Figure 1:**
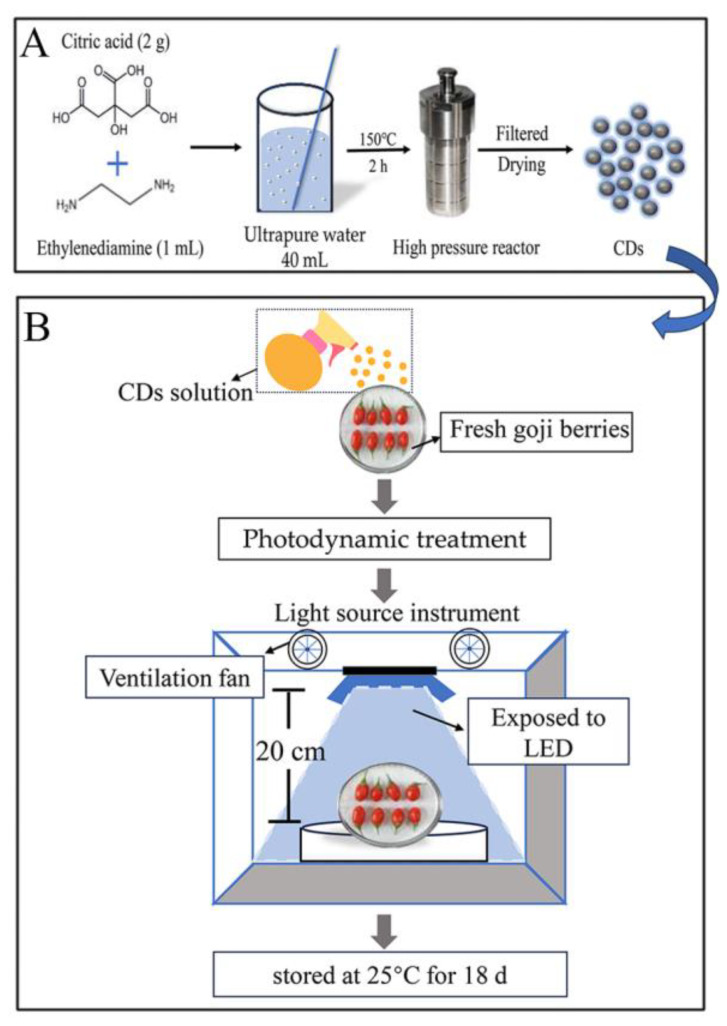
A schematic diagram of CD-mediated PDT treatment for fresh goji berries. (**A**) CD synthesis roadmap; (**B**) lighting information process diagram.

Based on the methods of Liu et al. [15], the synthesis method of CDs was improved and applied in this study (Figure 1A). Citric acid (2 g) was dissolved in 40 mL of ultrapure water, and then added to 1 mL of ethylenediamine (EDA). After completely dissolving the reagents in water, the mixture was autoclaved and then subjected to a follow-up treatment under the same conditions. Prior to centrifugation at 10,000 rpm for 15 min, the mixture was heated at 150 °C for 2 h followed by cooling at room temperature. Subsequently, the supernatant was filtered using a 0.2 µm filter element, and a consistent brown solution was obtained. Uniform particle sizes and a high quantum yield were ensured for the fluorescent CDs by transferring the solution (interception bag, 3000 Da) and purifying it at room temperature for 24 h. To end, the solution was frozen for 12 h and freeze dried to acquire solid CDs with favorable water solubility.

The synthesized CDs were characterized using a Teenai G2 F20 (FEI, Waltham, MA, USA) transmission electron microscope (TEM), FTIR spectrometer (Nicolet LS10, Waltham, MA, USA), and X-ray photoelectron spectrometer (XPS; Thermo escalab 250Xi, Waltham, MA, USA).

### 2.2. Cytotoxicity of Carbon Dots (CDs)

According to Ma et al. [27], for the biocompatibility of the prepared CDs, L929 fibroblasts were incubated at 37 °C for 24 h, transferred to a 96-well plate for an MTT (3-(4,5-Dimethylthiazol-2-yl)-2,5-diphenyltetrazolium bromide) assay, and exposed to CD solutions (0.05, 0.06, 0.07, 0.08, 0.09, 0.1, 0.11, and 0.12 g L^−1^) for 24 h. Untreated cells were used as the negative control, and the A490 and A630 were recorded for each well (TS630, Tokyo, Japan).

(1) $$\mathrm{Cell}\;\mathrm{viability}=1-\frac{C(A490-A630)\;-\;T(A490-A630)\;}{C(A490-A630)\;-\;B(A490-A630)}\times 100\%$$

A_C_, A_T_, and A_B_ represent the control group, drug treatment group, and blank control groups, respectively.

### 2.3. Experimental Design and Sample Treatment

The optimal concentration of the carbon dot (CDs) solution (0.1 g L^−1^), irradiation time (10 min), and light irradiance (100 mW cm^−2^) were obtained by conducting an orthogonal test (Supplementary Table S1) and range analysis (Table S3), and all the experimental groups were verified in terms of the appearance, decay rate, and weight loss rate.

The fresh fruit of *Lycium barbarum* L. cv. “Ningqi No. 1” were collected from the Goji Berry Garden, Nanliang Farm, Goji Berry Breeding Base, Goji Berry Research Institute of Ningxia Academy of Agriculture and Forestry Sciences (Yinchuan, Ningxia). After picking, fresh goji berries were transported to the laboratory within 1 h and immediately used for the experiments. The freshly picked goji berries were disinfected (0.1% sodium hypochlorite), washed with distilled water, and naturally air dried for later use. In the experiment (Figure 1B), blue light with wavelength of 450 nm was used as the light source for PDT in the treatment group (T group), and the blue light irradiance was fixed at 100 mW cm^−2^. Sixty samples of fresh goji berries were placed on the lifting platform 20 cm away from the light source, the CD solution with a concentration of 0.1 g L^−1^ was evenly sprayed on the surface of fresh goji berries and irradiated for 10 min, and the surface was turned over every 5 min to ensure uniform light reception. In the control group, all the processed samples were stored in a PVC freshness preservation box in the dark (25 °C, humidity 38%) for 18 d. From day 0, the decay rate, weight loss rate, and appearance changes in the fresh goji berries were observed every three days. The remaining fresh goji berries were stored under liquid nitrogen at −80 °C for the measurement of the other indexes.

### 2.4. Determination of Physical Properties

#### 2.4.1. Determination of Rotting Rate and Weight Loss Rate of Fresh Goji Berries

The rotting rate and weight loss rate of fresh goji berries were determined every 3 d through the method proposed Lv et al. [28], with modifications. The results were expressed using percentages. The experiment was repeated three times, and 60 fresh goji berries were used in each experiment. The fruit rotting rate was calculated as follows:

(2) $$\mathrm{Rotten}\;\mathrm{fruit}\;\mathrm{rate}\;=\frac{\mathrm{multiple}\;\mathrm{decayed}\;\mathrm{fruit}}{\mathrm{total}\mathrm{fruit}}\times 100\%$$

For the weight loss rate, the following formula was used:

(3) $$\mathrm{Weig}ht\;\mathrm{loss}\;=1-\frac{W_{0}-W_{t}}{W_{0}}\times 100\%$$

where W_0_ is the initial mass of the post-harvest fresh goji berries, and W_t_ is the mass during storage.

#### 2.4.2. Determination of Hardness of Fresh Goji Berries

Using a previous method described by Elam et al. [4], the firmness of the fresh goji berries was measured using texture analyzer (TMS-PRO physical analyzer, FTC Company, Boston, MA, USA) using the following parameters: pre-test speed of 100 mm/min, test speed of 30 mm/min, post-test speed of 100 mm/min, penetration distance of 5 mm, and trigger force of 0.05 N. The average value was obtained for each test of 10 berries, and the results were reported in Newtons (N).

#### 2.4.3. Determination of Color of Fresh Goji Berries

Based on the method of Zhang et al. [3], the a*, b*, L*, C*, and H* values of the peel color were calculated using a calibrated colorimeter (HP-200 colorimeter). L* indicated the brightness, [0 (black)–100 (white)], A* referred to red (+) to green (−), b* represented the yellow (+) to blue (−), the saturation *C* * = (a*^2^ + b*^2^)^1/2^, the hue angle H* = arctan (b*/a*), the total color change was represented by Δ*E*, the Δ*E* represented the color difference (L*, a*, and b*) of fresh goji berries on the Nth day of storage and the colors (*L*_0_*, *a*_0_*, and *b*_0_*) on the 0th day of storage, and the average value was obtained by measuring 10 berries each time.

(4) $$\Delta E=\sqrt{{\left(L^{*}-L_{0}^{*}\right)}^{2}+{\left(a^{*}-a_{0}^{*}\right)}^{2}+{\left(b^{*}-b_{0}^{*}\right)}^{2}}$$

### 2.5. Detection of Membrane Permeability and Active Oxygen Metabolism of Fresh Goji Berries

#### 2.5.1. Determination of Electrolyte Permeability

By using the method of Wang et al. [29] with modifications, the electrolyte permeability was determined. Fresh goji berries were cut into small disks (diameter of 0.5 cm, with 10 disks for 5 fresh goji berries) and washed with deionized water. The disks were transferred into a flask with 20 mL of deionized water and shaken gently on a shaker at 25 °C for 30 min to measure the electrolyte permeability (L_t_) of the solution. Then, the flask containing the solution was heated for 15 min (100 °C), quickly cooled, and measured (L_0_). Each assessment was repeated three times. The calculation method of electrolyte permeability was as follows:

(5) $$\mathrm{Electrolyte}\;\mathrm{permeability}\;=\;\frac{L_{t}}{L_{0}}\times 100\%$$

#### 2.5.2. Determination of Malondialdehyde (MDA)

The content of MDA was determined using an MDA content kit (Suzhou Comin Biotechnology Co., Ltd., Suzhou, China) [4]. Specifically, 0.1 g of the sample was weighed and added to a 1 mL extract for ice bath homogenization and then centrifuged (4 °C, 8000× *g*, 10 min) in a high-speed refrigerated centrifuge (3K30, Sigma Company, Shanghai, China). The supernatant was removed to react with thiobarbituric acid, and then the absorbance at 532 and 600 nm was determined with a multifunctional ELISA instrument (Mulotiska FC, Thermo Fisher Scientific, Shanghai, China). The calculated MDA concentration was expressed in μmol kg^−1^ (fresh weight).

#### 2.5.3. Determination of Hydrogen Peroxide (H_2_O_2_) Content and Superoxide Anion (O_2_^•−^) Production Rate

The production rates of H_2_O_2_ and O_2_^•−^ were determined to evaluate the production of reactive oxygen species by referring to the method of Elam et al. [4]. H_2_O_2_ content and O_2_^•−^ production rate kits (Suzhou Comin Biotechnology Co., Ltd., Suzhou, China) were used in the experiment, and the determination was performed in accordance with the instructions of the kits. The concentration of H_2_O_2_ was expressed in mmol kg^−1^, and the production rate of O_2_^•−^ was presented in mmol kg^−1^ min^−1^.

### 2.6. Determination of Quality in Fresh Goji Berries during Storage

#### 2.6.1. Determination of Total Soluble Solids (TSS), Titratable Acidity (TA), and Total Soluble Solids-to-Titratable Acidity Ratio (TSS/TA)

For the TSS and TA, a sugar and acid content analyzer (Sam-706 AC, G-Won High-Tech Co., Seoul, Korea) was used, and the TSS/TA ratio was calculated as shown in Lv et al. [28].

#### 2.6.2. Determination of Soluble Protein Content in Fresh Goji Berries during Storage

Coomassie Brilliant Blue (G-250), combined with protein to form a blue complex, was employed to evaluate the content of total protein in fresh goji berry samples [2]. In brief, 0.05 g of fresh goji berries tissue samples were weighed, added to 1 mL of distilled water for ice bath homogenization, and then centrifuged for 10 min (8000× *g*). Next, 100 μL of supernatant was removed and added to 100 μL of Coomassie Brilliant Blue solution to determine the absorbance at 620 nm, and the protein content was expressed in g kg^−1^.

#### 2.6.3. Determination of Total Phenol Content, Flavonoid Content, Total Polysaccharide Content, and Betaine Content in Fresh Goji Berries during Storage

The total phenols, flavonoids, betaines, and polysaccharides (phenol–sulfuric acid method) in fresh goji berries were determined using kits (Suzhou Comin Biotechnology Co., Ltd., Suzhou, China), and dried samples were used in the experiments in accordance with the kits’ instructions [2]. In brief, a multifunctional ELISA instrument was used to measure the blue compound produced by the reduction of tungstmolybdic acid with the use of phenols at 760 nm and further evaluate the total phenol content in the sample. First, a 0.1 g sample was weighed and added to a nitrite solution (2 mL) for an oscillation extraction at 60 °C for 2 h. Second, the crude extract was centrifuged (10,000× *g*) at 25 °C for 10 min. Finally, the supernatant was removed to measure the absorbance at 510 nm and calculate the flavonoid content in the sample. For the detection of the betaine content, 0.02 g of the sample was blended with 0.8 mL of distilled water. The mixture was then blended with a hydrochloric acid solution (200 µL) and centrifuged at room temperature (25 °C, 10,000× *g*) for 10 min. The supernatant was discarded, and the precipitate was kept. Subsequently, the precipitate was blended with acetone to measure the absorbance at 525 nm. The content of total polysaccharides was determined through the phenol–sulfuric acid method; that is, the content was determined by measuring the absorbance at 490 nm. All the above determination results were expressed in g kg^−1^.

#### 2.6.4. Determination of Carotenoid Content in Fresh Goji Berries during Storage

The method of Lv et al. [28] was referenced to determine the carotenoid content in fresh goji berries. The samples of fresh goji berries were dried (60 °C), crushed, and screened through a 100-mesh sieve. Roughly 1.0 g of dried samples were weighed in a mortar and ground evenly with a small amount of extracting solution (the volume ratio of absolute ethanol, acetone solution, and water was 4.5:4.5:1). Then, the volume of the extracting solution was fixed to 10 mL and extracted at 4 °C in the dark for 48 h, and the absorbance of the supernatant was measured at 440 nm.

(6) $$\mathrm{Carotenoid}\;({g\mathrm{kg}}^{-1})\;=\;\frac{A440\times V}{W}$$

### 2.7. Determination of Antioxidant Enzyme Activity and ASA–GSH Circulating System

#### 2.7.1. Determination of Antioxidant Enzyme Activity

The method proposed by Shi et al. [30] was referenced and slightly modified to evaluate the activities of antioxidant enzymes. During the experiment, the enzyme activities of catalase (CAT), superoxide dismutase (SOD), peroxidase (POD), phenylalanine ammonia-lyase (PAL), and polyphenol oxidase (PPO) in fresh goji berry samples were detected using the kits produced by Suzhou Comin Biotechnology Co., Ltd. Specifically, 1 mL of the extract provided in the kit was added into 0.1 g of the tissue sample, followed by ice bath homogenization. Then, the mixture was centrifuged (8000× *g*) at 4 °C for 10 min, and the supernatant was removed. Subsequently, the activities of CAT, SOD, POD, PPO, and PAL were determined in accordance with the instructions of the kits. The activities of all the enzymes were expressed in 10^6^ U kg^−1^ protein.

#### 2.7.2. Determination of Ascorbate–Glutathione Cycle

The contents of ascorbic acid (ASA) and glutathione (GSH) in the ASA–GSH circulating system were determined by reference to the research content of Ding et al. [31], which was slightly modified, and kits produced by Suzhou Comin Biotechnology Co., Ltd. were used. The experimental steps were carried out in accordance with the kits’ instructions. The concentrations of ASA and GSH were calculated by fresh weight, and the results were expressed in g kg^−1^ and mmol g^−1^, respectively.

The activities of ascorbate peroxidase (APX) and glutathione reductase (GR) enzymes in the ASA–GSH circulating system were detected in accordance with the previous research scheme of Wang et al. [2]. The experiment was performed using the kits produced by Suzhou Comin Biotechnology Co., Ltd. (Suzhou, China). Specifically, the activity of APX was calculated by determining the oxidation rate of AsA at 290 nm, and the oxidative amount in μmol of AsA per kilogram of protein per min was taken as 1 unit of enzyme activity (U). The change in the absorbance of nicotinamide adenine dinucleotide phosphate (NADPH) at 340 nm was determined to simplify the determination of the activity of GR, which reduced glutathione disulfide (GSSG) and then GSH by catalyzing NADPH; here, 1 unit of enzyme activity (U) was defined as the oxidative amount of 1 nmol NADPH as catalyzed by each kilogram of protein per min under alkaline conditions (pH = 8.0). The activities of APX and GR were expressed in 10^6^ U kg^−1^ protein.

### 2.8. Statistical Analysis

The results were expressed as means ± SD and subjected to an analysis of variance (ANOVA) using SPSS software (V29), using a *t*-test, where *p* < 0.05 * and *p* < 0.01 ** indicate significant and extremely significant differences, respectively.

## 3. Results and Discussion

### 3.1. Characterization of Carbon Dots (CDs)

**Figure 2:**
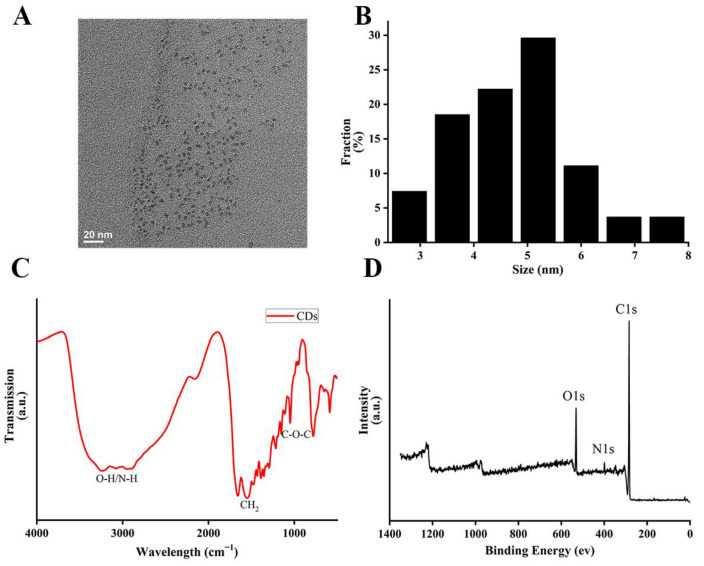
Characterization of physical properties of CDs. (**A**) TEM images of CDs; (**B**) size distribution of CDs; (**C**) FTIR spectrum of CDs; (**D**) XPS spectrum of CDs.

Figure 2A revealed that the hydrothermally synthesized CDs exhibited good dispersibility, with an average particle size of 4.66 nm (Figure 2B). According to Figure 2C, the peak at 3243 cm^−1^ is caused by an O–H/N–H stretching vibration in the carboxyl group of the CDs, the peak at 1549 cm^−1^ is caused by a N–H bending vibration, and the peak at 1044 cm^−1^ is related to the stretching vibration of C–O. These data show that CDs contain a large number of active groups such as hydroxyl, amino, and carboxyl groups. According to Figure 2D, the peaks at C1s (284 eV), O1s (531 eV), and N1s (400 eV) indicate the existence of carbon, oxygen, and nitrogen, which was consistent with the elemental analysis results (Supplementary Figure S1). Moreover, the CDs showed good biocompatibility without toxicity to L929 fibroblasts at the concentration of 0.1 g L^−1^ (Supplementary Figure S2). Therefore, CDs could be applied to PDT as the PS, along with having application potential in fruit preservation.

### 3.2. Optimal Treatment: Carbon Dot-Mediated Photodynamic Treatment of Fresh Goji Berries

**Figure 3:**
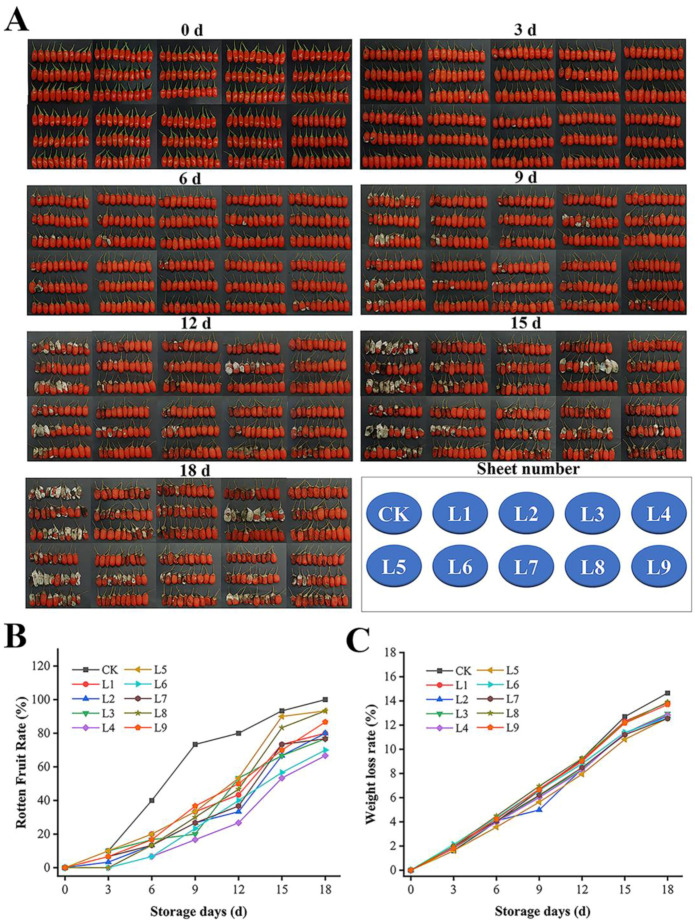
Optimal treatment: CD-mediated PDT of fresh goji berries. From 0 d to 18 d (**A**), rotten fruit rate (**B**), and weight loss rate (**C**). At 25 °C and a relative humidity of 38%, CK-L9 treatments are shown in Supplementary Table S2.

Through an orthogonal experimental design (Supplementary Table S2), for the CK, L1, L2, L3, L4, L5, L6, L7, L8, and L9, the appearance (Figure 3A), decay rate (Figure 3B), and weight loss rate (Figure 3C) of the nine treatment combinations were measured. Overall, the decay rate and weight loss rate of all treatment groups were lower compared to the control group, and the decay rate of L4 was the lowest, reaching 80% on the 18th day, which prolonged the storage period by 9 days. The observed appearance accorded with the range analysis result in the orthogonal experiment.

The main principle of PDT is that the PSs can generate ROS or active singlet oxygen (^1^O_2_) after being excited by a light source with a certain wavelength and then destroy biomolecules such as nucleic acids, soluble proteins, and lipids through oxidation, thus inactivating microorganisms or cells [32]. Many studies have shown that LED lighting without PSs cannot prevent the color change of fresh-cut papaya during storage at 4 °C [33]. Niu et al. [34] reported Fe_3_O_4_@Ce6/C6@silane nanoparticles (NPs) based on PDT, which exhibited excellent activity against Botrytis cinerea, while the control group without light irradiation exerted no inhibitory effect on Botrytis cinerea. Aponiene et al. [13] investigated the antibacterial effect of the combined treatment of visible light (405 nm), chlorophyll (Chl), and zinc oxide nanoparticles (NPs). The results showed that light alone had no effect on the activity of Escherichia coli, and only light sensitivity and zinc oxide NPs had negligible effects on the activity of Escherichia coli, but the photocatalytic treatment led to a significant decrease in the number of living cells. The “power” of CDs mainly depends on the concentration of CDs in the selected substrate and irradiation time [35]. Therefore, an effective PS combined with visible light, instead of the PS or light itself, is the key to the success of photodynamic therapy.

The CD concentrations, light irradiance, and irradiation time mainly affected the post-harvest storage period of fresh goji berries. Therefore, in the subsequent experiments, the harvested goji berry samples were irradiated under a fixed light irradiance of 100 mW cm^−2^ and a 0.1 g L^−1^ CD solution for 10 min.

### 3.3. Effect of Carbon Dot-Mediated Photodynamic Treatment on the Appearance Changes of Fresh Goji Berries

**Figure 4:**
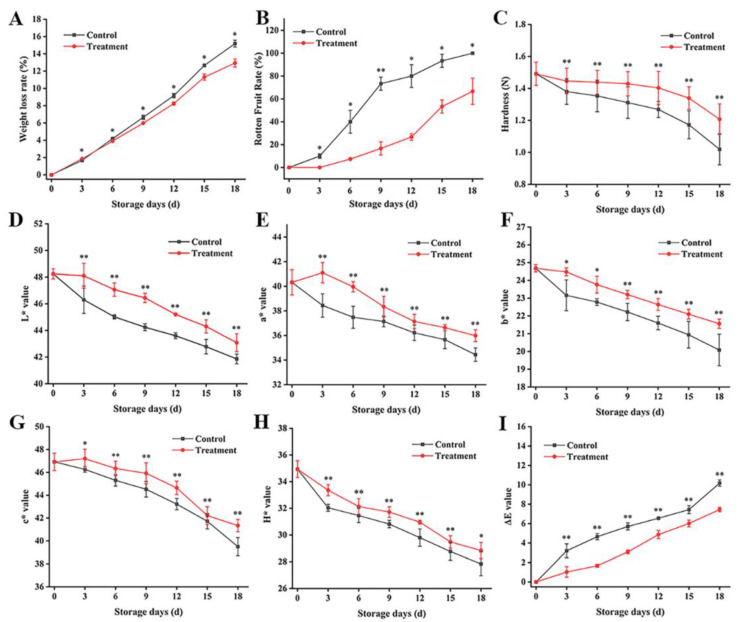
Effect of CD-mediated PDT on the appearance changes in fresh goji berries. The rotten fruit rate of fresh goji berries during storage period; weight loss rate; firmness; L* value; a* value; b* value; C* value; H* values; and Δ*E* values (**A**–**I**). The treatment group was treated with a 0.1 g L^−1^ CD solution for 10 min (light irradiance: 100 mW cm^−2^) and stored at 25 °C for 18 d. The control group was treated with distilled water. The data are expressed as the mean ± SD (standard deviation) (*n* = 3). Plots * and ** represent significant differences between the control and treatment group.

During the post-harvest storage of fruits and vegetables, the water loss in fruit increases with the storage time, during which the fruit are susceptible to microbial infection, leading to fruit softening and firmness decline. This phenomenon is mainly related to the decrease in cell wall components (cellulose, hemicellulose, and pectin), the polysaccharide molecular weight on the cell wall, and monosaccharide composition [36]. Overall, the weight loss rate (Figure 4A) of fresh goji berries increased, and the weight loss rate of the treatment group was always lower compared to control group. The decay rate (Figure 4B) also increased with the increase in storage time. From day 9, it was 50% lower compared to the control group, reaching 80% after 18 d, and the storage period increased by 9 d. The fruit firmness (Figure 4C) of fresh goji berries decreased during storage. From day 3, the fruit firmness of the treatment group was pointedly higher than that of the control group. Related studies have revealed that CDs have good antibacterial activity and generate more ROS (e.g., O_2_^•−^ and H_2_O_2_) through energy transfer after light excitation, causing damage and death to pathogens and reducing the decay index of the fruit during storage [35]. Du et al. [37] pointed out that CDs are highly targeting, do not damage the normal cells of fruit, and could serve as a free radical scavenger with an antioxidant effect. This finding supports that the CD-mediated PDT can effectively inhibit the water loss and decay index of fresh goji berries, slow down their firmness reduction, and prolong their post-harvest storage period.

Color change is also an important index that affects fruit quality. The L* (Figure 4D), a* (Figure 4E), b* (Figure 4F), C* (Figure 4G), and H* (Figure 4H) values of fresh goji berries subjected to the CD-mediated PDT and those in the control group both showed a declining trend, while the change in the total color difference Δ*E* (Figure 4H) during the storage period of fresh goji berries was significantly reduced. The change in the color index of fruit in the storage period could be expressed in terms of L*, in which a lower value represents a deeper fruit color [5]. Figure 3A shows that as the storage time was lengthened, all the fresh goji berries appeared darker, where the brightness change in the treatment group after 3 d was especially obvious and significantly lower. The fruit color parameter a* was influenced by the changes in anthocyanin, lycopene, and chlorophyll contents [38]. As shown in Figure 4E, the a* values for both the different groups showed a downward tendency, and that of the control group was significantly lower than of the treatment group, which was in consistency with the report in [34]. The color value is related to the content of carotenoids, which are responsible for the color of the fresh goji berries [39]. The results showed that the total color difference Δ*E* in fresh goji berries in the treatment group changed slowly. In a way, the CD-mediated PDT had the effect of maintaining the fruit color, which can be attributed to the high accumulation of carotenoids in fresh goji berries during storage.

### 3.4. Effects of Carbon Dot-Mediated Photodynamic Treatment on Membrane Permeability and Active Oxygen Metabolism of Fresh Goji Berries during Storage

**Figure 5:**
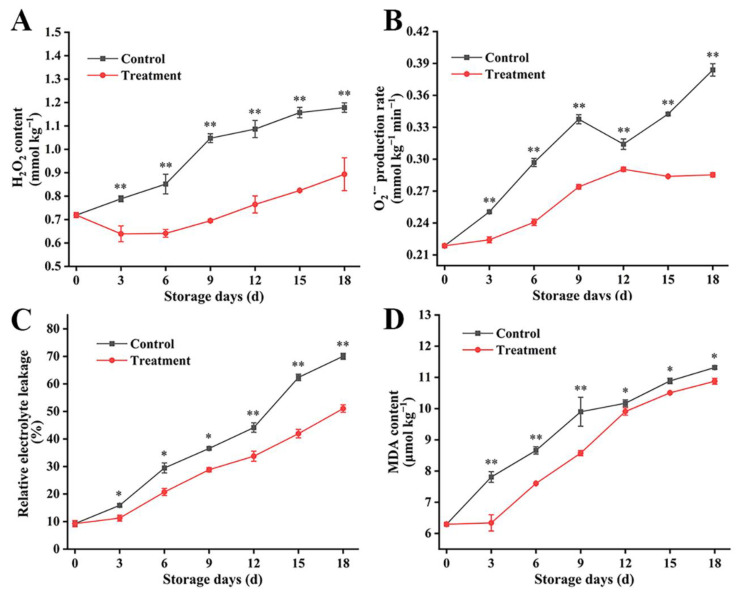
Effect of CD-mediated PDT of H_2_O_2_ content (**A**), O_2_^•−^ (**B**) electrolyte leakage, (**C**) and MDA content (**D**) in post-harvest fresh goji berries. The treatment group was treated with a 0.1 g L^−1^ CD solution for 10 min (light irradiance: 100 mW cm^−2^) and stored at 25 °C for 18 d. The control group was treated with distilled water. The data are expressed as the mean ± SD (standard deviation) (*n* = 3). Plots * and ** represent significant differences between the control and treatment group.

During post-harvest storage, the excessive production and accumulation of ROS accelerates the increase in lipid peroxidation and cell membrane permeability and damages the integrity of the cell membrane, thus accelerating the aging of fruit [40]. Electrolyte leakage and malondialdehyde (MDA) accumulation represent the degree of lipid peroxidation of the cell membrane and evaluate its integrity [41]. In this study, the content of active oxygen products hydrogen peroxide (H_2_O_2_) (Figure 5A) and superoxide anion (O_2_^•−^) (Figure 5B) increased significantly in the middle and late storage period, and the accumulated levels of O_2_^•−^and H_2_O_2_ in the control group were higher. During the whole storage period, the H_2_O_2_ content in the control group was 1.2 times more than that in the treatment group. The CD-mediated PDT also inhibited the accumulation of O_2_^•−^, especially in the late storage period, and the content of O_2_^•−^ in the treated fruit decreased by 15%. The electrolyte leakage (Figure 5C) increased in both the groups. After 15 and 18 days of storage, the electrolyte leakage in the control group was the largest, and the increasing trend of electrolyte leakage in fresh goji berries subjected to the CD-mediated PDT was significantly lower. Moreover, the content of MDA (Figure 5D) in all berries enlarged slowly with the storage time. High electrolyte leakage and MDA accumulation lead to the loss of cell membrane integrity and the peroxidation of membrane lipids, where the former can be attributed to the lack of intracellular adenosine triphosphate (ATP) and high ROS accumulation [42]. Therefore, a CD-mediated PDT can significantly reduce the accumulation of H_2_O_2_ and O_2_^•−^ in fresh goji berries and reduce the oxidative damage in the cell membrane, protecting the membrane integrity of fresh goji berries, thus improving the disease resistance of fruit and delaying the senescence of post-harvest fruit.

### 3.5. Effect of Carbon Dot-Mediated Photodynamic Treatment on the Quality Changes of Post-Harvest Fresh Goji Berries during Storage

**Figure 6:**
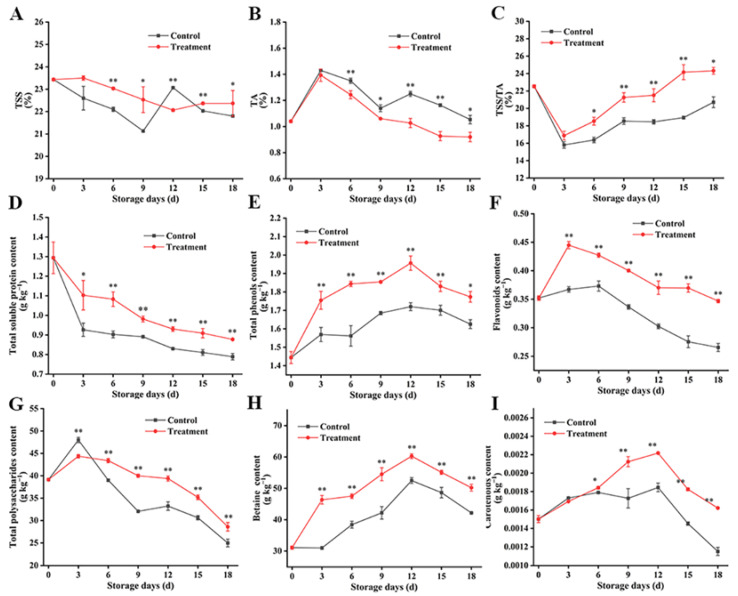
Effect of CD-mediated PDT of TSS content, TA content, TSS/TA content, total soluble protein content, total phenols, flavonoids, total polysaccharides, betaine total, and carotenoids in post-harvest fresh goji berries (**A**–**I**). The treatment group was treated with a 0.1 g L^−1^ CD solution for 10 min (light irradiance: 100 mW cm^−2^) and stored at 25 °C for 18 d. The control group was treated with distilled water. The data are expressed as the mean ± SD (standard deviation) (*n* = 3). Plots * and ** represent significant differences between the control and treatment group.

The composition of sugars and organic acids always changes dynamically in ripened fruit, which dominates the change law of fruit flavor and quality. The acidity of fleshy fruit measured by titratable acidity (TA) and the sugary state measured by the total soluble solids (TSS) are responsible for the sensory quality of fruit, and the TSS/TA value is an important index for the evaluation of the fruit quality [43,44]. Under the existence of respiration, the triggering of ethylene biosynthesis may not only coordinate the TSS/TA of fruit by regulating the metabolism of sugar and acid but also lead to fruit softening by promoting cell wall degradation after triggering the gene expressions and activities of PG and PME [45]. In the present study, the content of TSS (Figure 6A) in all samples began to decrease from day 3, and the content of TSS in the control group suddenly increased at 12 d. This phenomenon can be ascribed to the fruit softening in the late storage period. The content of TSS in the treatment group gradually decreased, with a light increase at day 15. The TA content first increased and then gradually decreased during storage (Figure 6B). The TA in the treatment group maintained a steady downward trend during the whole storage period. The results show that the CD-mediated PDT could slow down the decrease in TSS and the increase in TA in fresh goji berries, thus stabilizing the flavor of the fruit. Notably, the ratio of sugar to acid in both the groups reduced at first followed by an increase (Figure 6C). Zhang et al. [3] treated fresh goji berries with salicylic acid and thought that SA inhibited the respiration rate, ethylene production, and softening of fresh goji berries, leading to the decrease in the TA. Therefore, the CD-mediated PDT may also inhibit the respiration rate and ethylene production of fresh goji berries, thus delaying the increase in the TA. In a word, CD-mediated PDT can keep the high TSS and TSS/TA values and the low TA value of post-harvest fresh goji berries during storage, manifesting that the natural flavor of fresh goji berries can be effectively maintained by this treatment.

The quality of fresh goji berries deteriorates in the post-harvest stage because of its easy decay, thus seriously limiting their commercial value of fruit flavor and quality [1]. The CD-mediated PDT delayed the degradation of soluble protein (Figure 6D), total phenol (Figure 6E), flavonoid (Figure 6F), total polysaccharide (Figure 6G), betaine (Figure 6H), and carotenoid contents (Figure 6I) in fresh goji berries, and the soluble protein content was reduced with the storage time. However, the content of total phenols, flavonoids, total polysaccharides, betaine, and carotenoids in all goji berry samples first increased and then decreased, and the treated group showed consistently higher contents, in which the total content was twice as high as that of the control group. The content of total polysaccharides and flavonoids decreased slowly after reaching the peak after 3 d of storage, while the content of total phenols, betaine, and carotenoids decreased slowly after reaching the peak after 12 d of storage. Related studies have shown that in terms of the antioxidant capacity, the flavonoids of fresh goji berries have the strongest scavenging capacity for DPPH (1,1-diphenyl-2-picrylhydrazyl) and ABTS+ (2,2′-Azinobis-(3-ethylbenzthiazoline-6-sulphonate) free radicals, while polysaccharides have the strongest scavenging capacity for hydroxyl free radicals (•OH) and H_2_O_2_ [46]. In this study, a low weight loss rate and decay rate and high firmness were maintained in the treatment group of fresh goji berries during storage, because the nutritional content of the treatment group was better during storage, accompanied by the higher antioxidant capacity than that of the control group. Hence, the treated fresh goji berries were bright in color and full in particles during post-harvest storage, and the nutrients and antioxidant components of fresh goji berries remained stable. This finding is consistent with the conclusions regarding the chitosan coating [5], lecithin soaking [47], and salicylic acid soaking [3] of fresh goji berries. The results show that CD-mediated PDT delayed the loss of nutrients in fresh goji berries and kept their quality stable during storage.

### 3.6. Effects of Carbon Dot-Mediated Photodynamic Treatment on the Activity of the Antioxidant Enzyme System and ASA–GSH Circulating System

**Figure 7:**
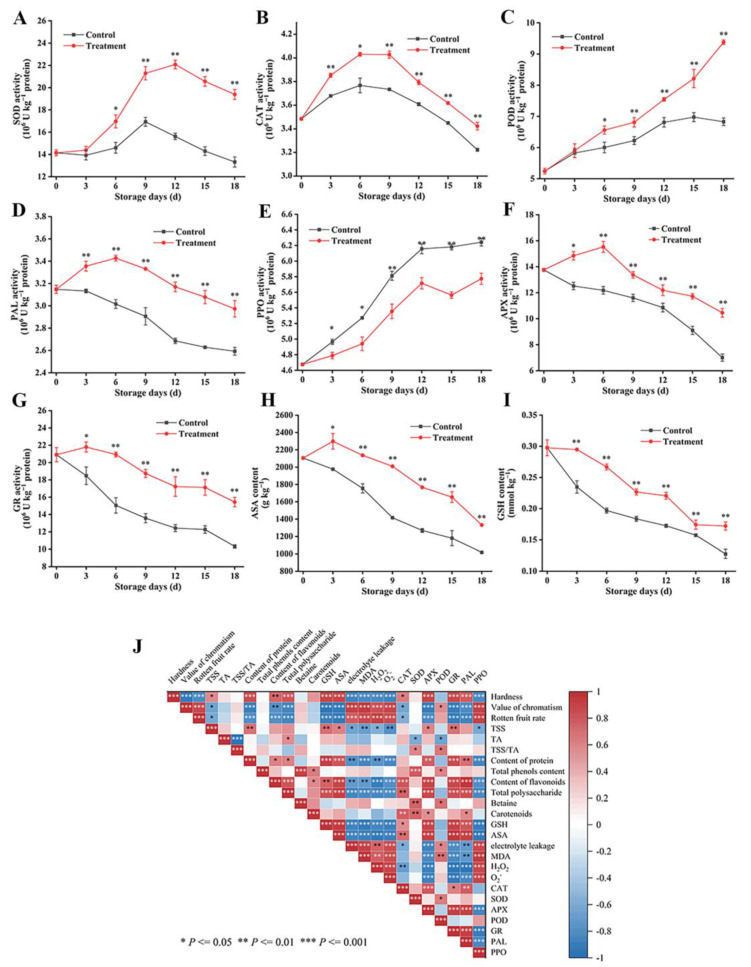
Effects of CD-mediated PDT on the activity of the antioxidant enzyme system and ASA–GSH circulating system in fresh goji berries during storage and the correlation analysis. Antioxidant enzyme activity: activity of SOD (**A**), CAT (**B**), POD (**C**), PAL (**D**), and PPO (**E**); ASA–GSH circulating system: activity of APX (**F**), GR (**G**), content of ASA (**H**), GSH (**I**), and correlation analysis (**J**). The treatment group was treated with a 0.1 g L^−1^ CD solution for 10 min (light irradiance: 100 mW cm^−2^) and stored at 25 °C for 18 d. The control group was treated with distilled water. The data are expressed as the mean ± SD (standard deviation) (*n* = 3). Plots * and ** represent significant differences between the control and treatment group.

In the life cycles of post-harvest fruits and vegetables, ROS are natural by-products of normal oxygen metabolism, which further increase with storage time, thus accelerating the quality deterioration of post-harvest fruits and vegetables, such as aging, browning, and softening [48]. A complete active oxygen scavenging system exists in fruits and vegetables, including various antioxidant enzymes and antioxidants. Therefore, improving the antioxidant enzymes and antioxidants can affect the balance of ROS and protecting cells [49]. Among them, superoxide dismutase (SOD), catalase (CAT), and peroxidase (POD) are the main antioxidant enzymes. During the whole storage period, compared with the control group, the CD-mediated PDT remarkably improved the activity of SOD (Figure 7A), CAT (Figure 7B), and POD (Figure 7C) of fresh goji berries. The SOD activities of the two groups of goji berry samples were improved followed by a decrease during the whole storage period. Moreover, the SOD activity of the treated group was always higher than that of the control group, with a significant difference from 6 d, and the SOD activity of the treated fresh goji berries increased by 30%. The CAT activity increased rapidly after 3 d and reached the peak after 9 d. The activity of POD showed an upward trend, and the activity of POD in the CD treatment group reached its peak after 9 d, and this value was twice higher. In addition, the activity of POD in the control group decreased after 12 d of storage. Research conducted by scientists has shown that CDs have potential as photosensitizers for PDT and do not quench singlet oxygen. They operate through a type II reaction mechanism [50], which was also confirmed by Nie et al. [51]. It is worth noting that CDs have demonstrated superior antibacterial efficacy compared to conventional small molecule photosensitizers (PS) [52]. In this study, the treatment group displayed reduced rot rates and electrolyte permeability during storage, along with a diminished accumulation of reactive oxygen species (ROS) in the fruit compared to the control group. Importantly, goji berry samples subjected to a PDT mediated by CDs exhibited elevated activities of SOD, CAT, and POD, which are beneficial for enhancing the fruit’s ability to scavenge ROS. The phenomenon of fruit disease resistance induced by a PDT mediated by CDs through a type II reaction mechanism, coupled with the enhancement of antioxidant enzyme activities, can be attributed to the preservation of the balance of ROS metabolism during storage and the retardation of the deterioration of fresh goji berries’ quality over time.

In the post-harvest life cycle, an increased phenylpropanoid pathway activity can promote bioactive compound accumulation. Niazi et al. [53] pointed out that the browning degree of persimmons during the post-harvest storage period can be significantly inhibited by a high phenylalanine ammonia-lyase (PAL) activity and low polyphenol oxidase (PPO) activity after persimmons were treated using hydrogen sulfide and γ-aminobutyric. During storage, the PAL activity of all samples increased, but the PPO activity decreased. Compared with the control fruit, the treated fresh goji berries showed a higher PAL activity (Figure 7D) and lower PPO activity (Figure 7E). Ohl et al. [54] found that PDT could regulate the expression of the PAL gene, and light could induce the endogenous PAL gene-coded transcript and the PAL-GUS (gene-fused glucuronidase; GUS) transcript. Tao et al. [55] showed that phenolic compounds rapidly accumulated in apples after PDT, which was also verified by Yu et al. [56]. This finding also revealed that PDT may enhance the generation of phenolic compounds. Therefore, CD-mediated PDT can promote the accumulation of phenols and flavonoids and reduce the accumulation of H_2_O_2_, O_2_^•−^, and MDA by alleviating the decrease in PAL activity and the increase in PPO activity in fresh goji berries during storage. A possible reason is that a CD-mediated PDT could effectively prevent the loss of antioxidants such as phenolic compounds and strengthen the intracellular ROS scavenging ability of fruit by keeping the integrity of the protective film.

The AsA–GSH cycle plays a key role in maintaining the redox homeostasis of cells [57]. As an electron donor of ascorbate peroxidase (APX), AsA catalyzes the transformation of intracellular H_2_O_2_ into H_2_O, which is the main antioxidant enzyme for scavenging H_2_O_2_ in plant tissues and cells. Glutathione reductase (GR), which is a flavoprotein oxidoreductase, reduces oxidized glutathione (GSSG) to GSH by using NADPH, provides sufficient glutathione (GSH) for dehydroascorbic acid reductase, and reduces dehydroascorbic acid to AsA [58]. Therefore, the high activities of APX and GR are of importance for the accumulation of AsA and GSH. In this study, compared with the control group, the fresh goji berries subjected to the CD-mediated PDT had a higher accumulation of AsA (Figure 7H) and GSH (Figure 7I), and this process is accompanied by the increase in GR (Figure 7G) and APX (Figure 7F). Wang et al. [59] thought that salicylic acid could promote the accumulation of AsA and GSH by improving the activity of the APX/GR system in peaches. Zhu et al. [60] treated peaches with methyl jasmonate and scavenged ROS by increasing the content of AsA and GSH, indicating that AsA–GSH was an important mechanism to maintain the steady state and redox state of ROS. In summary, in this study, fresh goji berries treated with a CD-mediated PDT promoted the accumulation of AsA and GSH by increasing the activity of GR and provided APX with electron donors to inhibit the accumulation of H_2_O_2_ in fresh goji berries, thus protecting post-harvest fresh goji berries from oxidative damage and delaying their senescence. In a word, in the ROS scavenging system, SOD is the first line of defense, followed by APX, CAT, POD, and GR. The CD-mediated PDT can inhibit the accumulation of H_2_O_2_ and O_2_^•−^, reduce the damage of ROS, and delay post-harvest senescence by improving SOD, CAT, POD, GR, and APX levels and increasing the accumulation of non-enzymatic antioxidants.

According to the Pearson correlation coefficient, correlation analyses between the quality indexes and antioxidant enzymes of fresh goji berries during storage are shown in Figure 7J. The fruit firmness was positively correlated with soluble solids, soluble protein, flavonoids, total polysaccharides, ascorbic acid, GSH, AsA, GR, PAL, CAT, SOD, and APX activity, and it was negatively correlated with the peel color difference, fruit decay rate, electrolyte leakage rate, MDA, H_2_O_2_, O_2_^•−^, and PPO activity. Notably, peel browning was negatively correlated with the activities of the PAL, GR, and APX enzymes accumulated by AsA and GSH, whereas peel browning was positively correlated with the accumulation of O_2_^•−^, H_2_O_2_, MDA, and PPO enzymatic activity. Fruit hardness was also negatively correlated with the accumulation of O_2_^•−^, H_2_O_2_, MDA, and PPO enzymatic activity.

## 4. Conclusions

The excitation of 0.1 g L^−1^ of CD solution at 450 nm can significantly reduce the decay degree of fresh goji berries during storage, prolong the shelf life by 9 d, and maintain the edible value and sensory quality of fresh goji berries. Enhancing the activities of SOD, CAT, APX, POD, and GR can promote the accumulation of AsA and GSH antioxidants, reduce the accumulation of MDA, H_2_O_2_, and O_2_^•−^, and scavenge ROS. Meanwhile, increasing the activity of PAL and reducing that of PPO can strengthen the accumulation of phenols and flavonoids, relieve fruit browning, and delay fruit senescence. As a new-type, post-harvest storage method of fresh goji berries, CD-mediated PDT has broad development and application prospects in the field of fruit and vegetable preservation.

## Data Availability

The original contributions presented in the study are included in the article and Supplementary Materials, further inquiries can be directed to the corresponding authors.

## Supplementary Materials

The following supporting information can be downloaded at: https://www.mdpi.com/article/10.3390/foods13060955/s1, Table S1: Factors and factor levels of orthogonal experiment; Table S2: Table of orthogonal experiments and experimental data; Table S3: Range analysis results; Table S4: Analysis of variance table; Figure S1: Elemental spectra of C1s, N1s, and O1s in CDs. Figure S2: Cytotoxicity analysis of the CDs towards L929 mouse fibroblast cell lines when exposed with varying concentrations for a 24 h period.

## References

[1] Ma R.H., Zhang X.X., Thakur K., Zhang J.G., Wei Z.J. (2022). Research progress of *Lycium barbarum* L. as functional food: Phytochemical composition and health benefits. Curr. Opin. Food Sci..

[2] Wang W., Ni Z.J., Song C.B., Ma W.P., Cao S.Q., Wei Z.J. (2023). Hydrogen sulfide treatment improves quality attributes via regulating the antioxidant system in goji berry (*Lycium barbarum* L.). Food Chem..

[3] Zhang H.Y., Ma Z.M., Wang J.J., Wang P., Lu D.Y., Deng S.F., Lei H.L., Gao Y.F., Tao Y.Y. (2021). Treatment with exogenous salicylic acid maintains quality, increases bioactive compounds, and enhances the antioxidant capacity of fresh goji (*Lycium barbarum* L.) fruit during storage. LWT.

[4] Elam E., Lv Y.M., Wang W., Thakur K., Ma W.P., Ni Z.J., Wei Z.J. (2022). Effects of nitric oxide on postharvest storage quality of *Lycium barbarum* fruit. Food Sci. Technol..

[5] Ban Z.J., Wei W.W., Yang X.Z., Feng J.H., Guan J.F., Li L. (2015). Combination of heat treatment and chitosan coating to improve postharvest quality of wolfberry (*Lycium barbarum*). Int. J. Food. Sci. Technol..

[6] Fan X.J., Zhang B., Yan H., Feng J.T., Ma Z.Q., Zhang X. (2019). Effect of lotus leaf extract incorporated composite coating on the postharvest quality of fresh goji (*Lycium barbarum* L.) fruit. Physiol. Plant.

[7] Liu D., Gu W.M., Wang L., Sun J.X. (2023). Photodynamic inactivation and its application in food preservation. Crit. Rev. Food. Sci. Nutr..

[8] Buchovec I., Lukseviciūtė V., Kokstaite R., Labeikyte D., Kaziukonyte L., Luksiene Z. (2017). Inactivation of Gram (-) bacteria Salmonella enterica by chlorophyllin-based photosensitization: Mechanism of action and new strategies to enhance the inactivation efficiency. J. Photochem. Photobiol. B.

[9] Bacellar I.O.L., Baptista M.S. (2019). Mechanisms of photosensitized lipid oxidation and membrane permeabilization. ACS Omega.

[10] Sun Y.N., Zhang M., Bhandari B., Yang C.H. (2022). Recent development of carbon quantum dots: Biological toxicity, antibacterial properties and application in foods. Food Rev. Int..

[11] Maisch T., Eichner A., Späth A., Gollmer A., König B., Regensburger J., Bäumler W. (2014). Fast and effective photodynamic inactivation of multiresistant bacteria by cationic riboflavin derivatives. PLoS ONE.

[12] Penha C.B., Bonin E., da Silva A.F., Hioka N., Zanqueta É.B., Nakamura T.U., de Abreu Filho B.A., Campanerut-Sá P.A.Z., Mikcha J.M.G. (2017). Photodynamic inactivation of foodborne and food spoilage bacteria by curcumin. LWT.

[13] Aponiene K., Paskeviciute E., Reklaitis I., Luksiene Z. (2015). Reduction of microbial contamination of fruits and vegetables by hypericin-based photosensitization: Comparison with other emerging antimicrobial treatments. J. Food Eng..

[14] Ghate V.S., Zhou W.B., Yuk H.G. (2019). Perspectives and Trends in the Application of Photodynamic Inactivation for Microbiological Food Safety. Compr. Rev. Food Sci. Food Saf..

[15] Liu S.H., Cui J.L., Huang J.B., Tian B.S., Jia F., Wang Z.L. (2019). Facile one-pot synthesis of highly fluorescent nitrogen-doped carbon dots by mild hydrothermal method and their applications in detection of Cr(VI) ions. Spectrochim. Acta Part A Mol. Biomol. Spectrosc..

[16] Wen F.Z., Li P.Y., Yan H.J., Su W. (2023). Turmeric carbon quantum dots enhanced chitosan nanocomposite films based on photodynamic inactivation technology for antibacterial food packaging. Carbohydr. Polym..

[17] Liu Y.S., Zhao Y.N., Zhang Y.Y. (2014). One-step green synthesized fluorescent carbon nanodots from bamboo leaves for copper(II) ion detection. Sens. Actuators B Chem..

[18] Wang J., Wang C.F., Chen S. (2012). Amphiphilic egg-derived carbon dots: Rapid plasma fabrication, pyrolysis process, and multicolor printing patterns. Angew. Chem. Int. Edit..

[19] Song Y.B., Zhu S.J., Zhang S.T., Fu Y.Y., Wang L., Zhao X.H., Yang B. (2015). Investigation from chemical structure to photoluminescent mechanism: A type of carbon dots from the pyrolysis of citric acid and an amine. J. Mater. Chem. C.

[20] Zhai X.Y., Zhang P., Liu C.J., Bai T., Li W.C., Dai L.M., Liu W.G. (2012). Highly luminescent carbon nanodots by microwave-assisted pyrolysis. ChemComm.

[21] Wang B.G., Tang W.W., Lu H.S., Huang Z.Y. (2015). Hydrothermal synthesis of ionic liquid-capped carbon quantum dots with high thermal stability and anion responsiveness. J. Mater. Sci..

[22] Liu H.X., Zhong X., Pan Q., Zhang Y., Deng W.T., Zou G.Q., Hou H.S., Ji X.B. (2024). A review of carbon dots in synthesis strategy. Coord. Chem. Rev..

[23] Fan K., Zhang M., Fan D.C., Jiang F.J. (2019). Effect of carbon dots with chitosan coating on microorganisms and storage quality of modified-atmosphere-packaged fresh-cut cucumber. J. Sci. Food. Agric..

[24] Riahi Z., Rhim J.-W., Bagheri R., Pircheraghi G., Lotfali E. (2022). Carboxymethyl cellulose-based functional film integrated with chitosan-based carbon quantum dots for active food packaging applications. Prog. Org. Coat..

[25] Wang X., Zhao L., Hu J.S., Wei H., Liu X.Y., Li E.S., Yang S.H. (2022). Rational design of novel carbon-oxygen quantum dots for ratiometrically mapping pH and reactive oxygen species scavenging. Carbon.

[26] Zhu S.J., Meng Q.N., Wang L., Zhang J.H., Song Y.B., Jin H., Zhang K., Sun H.C., Wang H.Y., Yang B. (2013). Highly photoluminescent carbon dots for multicolor patterning, sensors, and bioimaging. Angew. Chem. Int. Edit..

[27] Ma R.H., Ni Z.J., Zhang F., Zhang Y.Y., Liu M.M., Thakur K., Zhang J.G., Wang S., Wei Z.J. (2020). 6-Shogaol mediated ROS production and apoptosis via endoplasmic reticulum and mitochondrial pathways in human endometrial carcinoma Ishikawa cells. J. Funct..

[28] Lv Y.M., Elnur E., Wang W., Thakur K., Du J., Li H.N., Ma W.P., Liu Y.Q., Ni Z.J., Wei Z.J. (2022). Hydrogen sulfide treatment increases the antioxidant capacity of fresh Lingwu Long Jujube (*Ziziphus jujuba* cv. Mill) fruit during storage. Curr. Res. Food Sci..

[29] Wang Y.J., Li Y.X., Yang S.H., Li C., Li L., Gao S.Y., Wu Z.X. (2023). Mechanism of ozone treatment in delayed softening of fresh-cut kiwifruit during storage. Postharvest Biol. Technol..

[30] Shi J.K., Xie W.X., Li S.N., Wang Y., Wang Q.G., Li Q.Q. (2024). Prohibitin StPHB3 affects the browning of fresh-cut potatoes via influencing antioxidant capacity and polyphenol oxidase activation. Postharvest Biol. Technol..

[31] Ding X.C., Liu S., Duan X.W., Pan X.J., Dong B.Y. (2023). MAPK cascade and ROS metabolism are involved in GABA-induced disease resistance in red pitaya fruit. Postharvest Biol. Technol..

[32] Correia J.H., Rodrigues J.A., Pimenta S., Dong T., Yang Z. (2021). Photodynamic therapy review: Principles, photosensitizers, applications, and future directions. Pharmaceutics.

[33] Kim M.J., Bang W.S., Yuk H.G. (2017). 405 ± 5 nm light emitting diode illumination causes photodynamic inactivation of *Salmonella* spp. on fresh-cut papaya without deterioration. Food Microbiol..

[34] Niu X.D., Liu L., Wang H.S., Lin L., Yang Y.A., Gao Y.W., Wang X.Y., Sun J., Dong B. (2021). Discovery of novel photosensitized nanoparticles as a preservative for the storage of strawberries and their activity against *Botrytis cinerea*. LWT.

[35] Knoblauch R., Geddes C.D. (2020). Carbon nanodots in photodynamic antimicrobial therapy: A review. Materials.

[36] Chea S., Yu D.J., Park J., Oh H.D., Chung S.W., Lee H.J. (2019). Fruit softening correlates with enzymatic and compositional changes in fruit cell wall during ripening in ‘Bluecrop’ highbush blueberries. Sci. Hortic..

[37] Du F.F., Shuang S.M., Guo Z.H., Gong X.J., Dong C., Xian M., Yang Z.H. (2020). Rapid synthesis of multifunctional carbon nanodots as effective antioxidants, antibacterial agents, and quercetin nanoprobes. Talanta.

[38] Li D., Zhang X.C., Li L., Aghdam M.S., Wei X.X., Liu J.Q., Xu Y.Q., Luo Z.S. (2019). Elevated CO_2_ delayed the chlorophyll degradation and anthocyanin accumulation in postharvest strawberry fruit. Food Chem..

[39] Patsilinakos A., Ragno R., Carradori S., Petralito S., Cesa S. (2018). Carotenoid content of Goji berries: CIELAB, HPLC-DAD analyses and quantitative correlation. Food Chem..

[40] Ding Z.S., Tian S.P., Zheng X.L., Zhou Z.W., Xu Y. (2007). Responses of reactive oxygen metabolism and quality in mango fruit to exogenous oxalic acid or salicylic acid under chilling temperature stress. Physiol. Plant.

[41] Luo Z., Chen C., Xie J. (2011). Effect of salicylic acid treatment on alleviating postharvest chilling injury of ‘Qingnai’ plum fruit. Postharvest Biol. Technol..

[42] Zhao Q.X., Jin M.J., Guo L.Y., Pei H.H., Nan Y.Y., Rao J.P. (2020). Modified atmosphere packaging and 1-methylcyclopropene alleviate chilling injury of ‘Youhou’ sweet persimmon during cold storage. Food Packag. Shelf Life.

[43] Nishizawa A., Yabuta Y., Shigeoka S. (2008). Galactinol and raffinose constitute a novel function to protect plants from oxidative damage. Plant Physiol..

[44] Chen Y.H., Sun J.Z., Lin H.T., Hung Y.C., Zhang S., Lin Y.F., Lin T. (2017). Paper-based 1-MCP treatment suppresses cell wall metabolism and delays softening of Huanghua pears during storage. J. Sci. Food Agric..

[45] Zhao J.H., Li H.X., Xi W.P., An W., Niu L.L., Cao Y.L., Wang H.F., Wang Y.J., Yin Y. (2015). Changes in sugars and organic acids in wolfberry (*Lycium barbarum* L.) fruit during development and maturation. Food Chem..

[46] Wang C.C., Chang S.C., Inbaraj B.S., Chen B.H. (2010). Isolation of carotenoids, flavonoids and polysaccharides from *Lycium barbarum* L. and evaluation of antioxidant activity. Food Chem..

[47] Jatoi M.A., Jurić S., Vidrih R., Vinceković M., Vuković M., Jemrić T. (2017). The effects of postharvest application of lecithin to improve storage potential and quality of fresh goji (*Lycium barbarum* L.) berries. Food Chem..

[48] Meitha K., Pramesti Y., Suhandono S. (2020). Reactive oxygen species and antioxidants in postharvest vegetables and fruits. Int. J. Food Sci..

[49] Huan C., Han S., Jiang L., An X.J., Yu M.L., Xu Y., Ma R.J., Yu Z.F. (2017). Postharvest hot air and hot water treatments affect the antioxidant system in peach fruit during refrigerated storage. Postharvest Biol. Technol..

[50] Stanković N.K., Bodik M., Šiffalovič P., Kotlar M., Mičušik M., Špitalsky Z., Danko M., Milivojević D.D., Kleinova A., Kubat P. (2018). Antibacterial and antibiofouling properties of light triggered fluorescent hydrophobic carbon quantum dots langmuir–blodgett thin films. ACS Sustain. Chem. Eng..

[51] Nie X.L., Jiang C.Y., Wu S.L., Chen W.B.F., Lv P.F., Wang Q.Q., Liu J.Y., Narh C., Cao X.M., Ghiladi R.A. (2020). Carbon quantum dots: A bright future as photosensitizers for in vitro antibacterial photodynamic inactivation. J. Photochem..

[52] Zhang J.Y., Lu X.M., Tang D.D., Wu S.H., Hou X.D., Liu J.W., Wu P. (2018). Phosphorescent carbon dots for highly efficient oxygen photosensitization and as photo-oxidative nanozymes. ACS Appl. Mater. Interfaces.

[53] Niazi Z., Razavi F., Khademi O., Aghdam M.S. (2021). Exogenous application of hydrogen sulfide and γ-aminobutyric acid alleviates chilling injury and preserves quality of persimmon fruit (*Diospyros kaki*, cv. Karaj) during cold storage. Sci. Hortic..

[54] Ohl S., Hedrick S.A., Chory J., Lamb C.J. (1990). Functional properties of a phenylalanine ammonia-lyase promoter from Arabidopsis. Plant Cell.

[55] Tao R., Zhang F., Tang Q.J., Xu C.S., Ni Z.J., Meng X.H. (2019). Effects of curcumin-based photodynamic treatment on the storage quality of fresh-cut apples. Food Chem..

[56] Yu J.S., Zhang F., Zhang J., Han Q.M., Song L.L., Meng X.H. (2021). Effect of photodynamic treatments on quality and antioxidant properties of fresh-cut potatoes. Food Chem..

[57] Davey M.W., Montagu M.V., Inzé D., Sanmartin M., Kanellis A., Smirnoff N., Benzie I.J.J., Strain J.J., Favell D., Fletcher J. (2000). Plant L-ascorbic acid: Chemistry, function, metabolism, bioavailability and effects of processing. J. Sci. Food Agric..

[58] Dokhanieh A.Y., Aghdam M.S., Fard J.R., Hassanpour H. (2013). Postharvest salicylic acid treatment enhances antioxidant potential of cornelian cherry fruit. Sci. Hortic..

[59] Wang L.J., Chen S.J., Kong W.F., Li S.H., Archbold D.D. (2006). Salicylic acid pretreatment alleviates chilling injury and affects the antioxidant system and heat shock proteins of peaches during cold storage. Postharvest Biol. Technol..

[60] Zhu L.J., Yu H.T., Dai X.M., Yu M.L., Yu Z.F. (2022). Effect of methyl jasmonate on the quality and antioxidant capacity by modulating ascorbate-glutathione cycle in peach fruit. Sci. Hortic..

