# Supplementary material for: Carbon Dot-Mediated Photodynamic Treatment Improves the Quality Attributes of Post-Harvest Goji Berries (Lycium barbarum L.) via Regulating the Antioxidant System
Source: Foods. 2024 Mar 21;13(6):955. doi: 10.3390/foods13060955 (PMC10970019; doi:10.3390/foods13060955)
Supplement: Supplementary file 1 [file foods-13-00955-s001.zip › foods-2915479-supplementary.pdf]

Supplementary Table S1: Factors and factor levels of orthogonal experiment

Levels	Factors		
	A-Concentration ($g\ L^{-1}$)	B-Light irradiance ($mW\ cm^{-2}$)	C-Time (min)
1	0.05	100.00	5.00
2	0.10	125.00	10.00
3	0.15	145.00	15.00

Supplementary Table S2: Table of orthogonal experiments and experimental data.

Experimental serial numbe	Factors and levels			Experimental indicators
	Concentration (g L^{-1})	Light irradiance (mW cm^{-2})	Time (min)	Rotting rate (%)
1	0.05	100.00	5.00	83.33
2	0.05	125.00	10.00	89.67
3	0.05	145.00	15.00	95.33
4	0.10	100.00	10.00	70.00
5	0.10	125.00	15.00	79.33
6	0.10	145.00	5.00	86.67
7	0.15	100.00	15.00	88.00
8	0.15	125.00	5.00	93.33
9	0.15	145.00	10.00	96.67

Supplementary Table S3: Range analysis results

Experimental serial numbe	A- Concentration	B- Light irradiance	C-Time
K_1	268.33	241.33	263.33
K_2	236.00	262.33	256.34
K_3	278.00	278.67	262.66
k_1	89.44	80.44	87.78
k_2	78.67	87.44	85.45
k_3	92.67	92.89	87.55
r	14.00	12.45	2.33
Main and Secondary Factor: A > B > C Optimal combination: A2B1C2			

Supplementary Table S4: Analysis of variance table.

Factors	Sum of squares of deviation	Degree of freedom	Mean square deviation	F value	P value	significant difference
A	322.53	2.00	161.26	50.21	0.02	*
B	233.59	2.00	116.79	36.36	0.03	*
C	9.92	2.00	4.96	1.54	0.39	
Error	6.42	2.00	3.21			
Total	527.45	9.00	58.61			

Note: * * indicates a highly significant (P<0.01) impact, and * indicates a significant (P<0.05) impact.

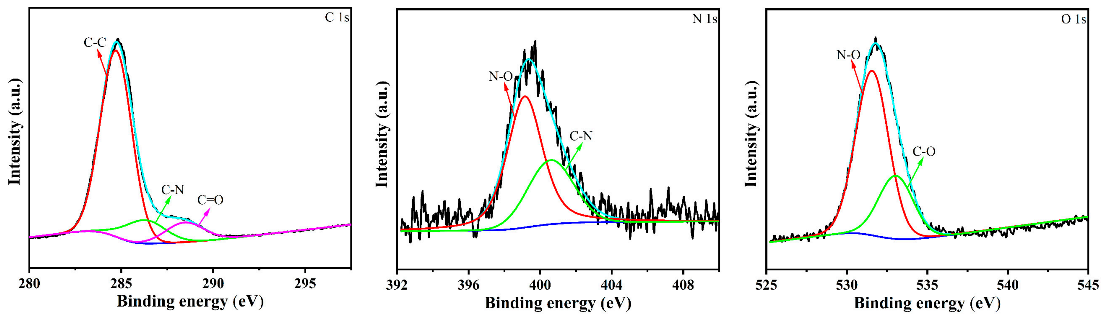

Supplementary Figure S1: Elemental spectra of C1s, N1s, and O1s in CDs.

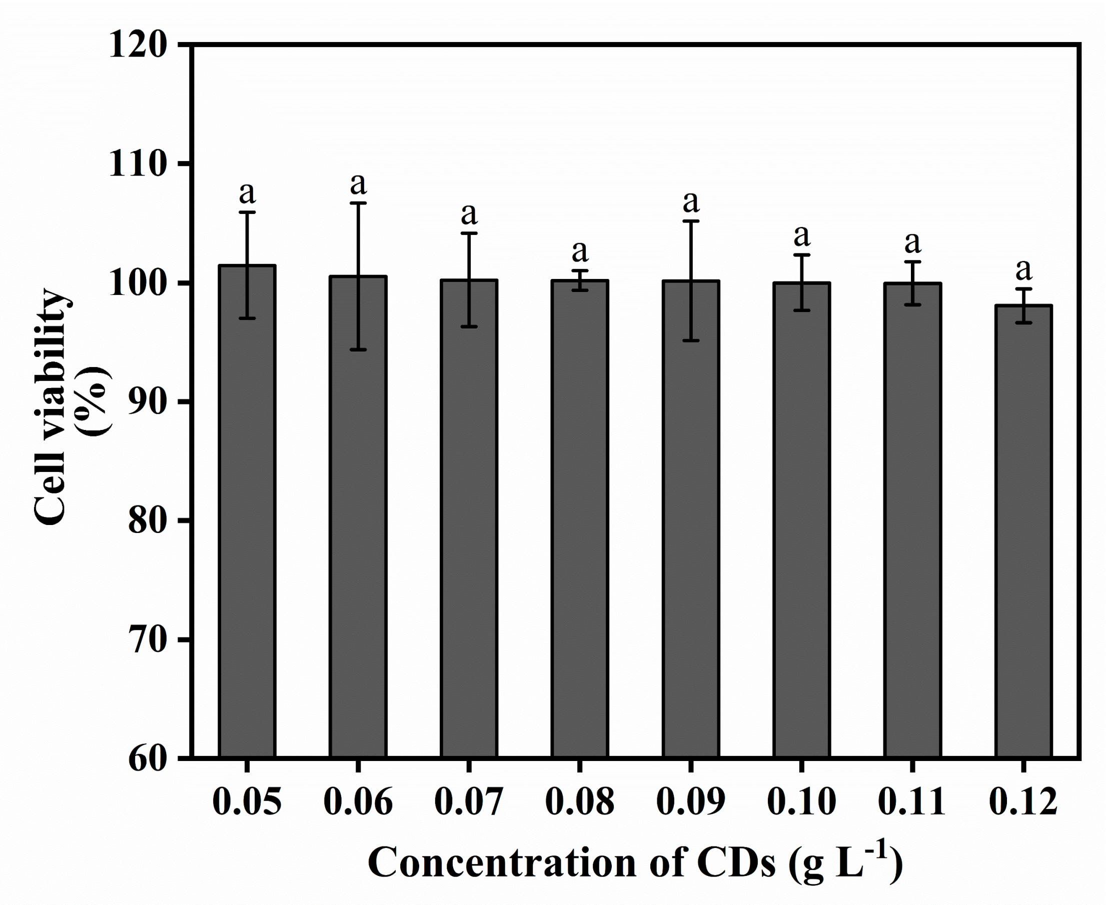

Supplementary Figure S2: Cytotoxicity analysis of the CDs towards L929 mouse fibroblast cell lines when exposed with varying concentrations for a 24 h period. Superscript letters of a-d in the same line represent significant differences estimated by Duncan's multiple range test ($P<0.05$)
